# Exploring the multiverse of analysis options for the alcohol Stroop

**DOI:** 10.3758/s13428-024-02377-5

**Published:** 2024-03-14

**Authors:** Andrew Jones, Elena Petrovskaya, Tom Stafford

**Affiliations:** 1 School of Psychology, Tom Reilly Building, Liverpool John Moore’s University, Byrom Street, L3 3AF Liverpool, UK; 2 Department of Computer Science, University of York, York, UK; 3 Department of Psychology, University of Sheffield, Sheffield, UK

**Keywords:** Alcohol Stroop, Alcohol, Craving, Multiverse, Specification curve analysis

## Abstract

The alcohol Stroop is a widely used task in addiction science to measure the theoretical concept of attentional bias (a selective attention to alcohol-related cues in the environment), which is thought to be associated with clinical outcomes (craving and consumption). However, recent research suggests findings from this task can be equivocal. This may be because the task has many different potential analysis pipelines, which increase researcher degrees of freedom when analysing data and reporting results. These analysis pipelines largely come from how outlying reaction times on the task are identified and handled (e.g. individual reaction times > 3 standard deviations from the mean are removed from the distribution; removal of all participant data if > 25% errors are made). We used specification curve analysis across two alcohol Stroop datasets using alcohol-related stimuli (one published and one novel) to examine the robustness of the alcohol Stroop effect to different analytical decisions. We used a prior review of this research area to identify 27 unique analysis pipelines. Across both data sets, the pattern of results was similar. The alcohol Stroop effect was present and largely robust to different analysis pipelines. Increased variability in the Stroop effect was observed when implementing outlier cut-offs for individual reaction times, rather than the removal of participants. Stricter outlier thresholds tended to reduce the size of the Stroop interference effect. These specification curve analyses are the first to examine the robustness of the alcohol Stroop to different analysis strategies, and we encourage researchers to adopt such analytical methods to increase confidence in their inferences across cognitive and addiction science.

## Introduction

Over the past decade, there has been increased awareness and discussion of a ‘*reproducibility crisis* or *renaissance’* within science, and in particular psychology (Nosek et al., 2022). Many seemingly robust research findings have since failed to replicate or have come under increased scrutiny from the field. For example, in 2015 the Open Science Collaboration attempted to replicate 100 psychology studies published in high-impact journals (Open Science Collaboration, 2015), with increased statistical power. Only 36% of the studies were replicated and overall, the effect sizes from the replications were much smaller than those reported in the original studies. Subsequent large-scale replication attempts have also demonstrated a similar pattern (see Hagger et al., 2016; Klein et al., 2014). Whilst it is extremely difficult to identify the cause of unreliable findings, researchers have suggested it may be a combination of low-powered studies (Button et al., 2013), questionable research practices (Xie et al., 2021), poor transparency in reporting (Simmons et al., 2011), and opportunistic use of researcher degrees of freedom (Wicherts et al., 2016).

Researcher degrees of freedom are the numerous, often defensible, but arbitrary choices that are made when collecting, analysing, and reporting data for an experiment. This includes determining when to stop collecting data, how to randomise participants, how to identify and handle outlying data points, and which variable to select as a primary outcome (see Wicherts et al., 2016). In each of these cases, a decision will need to be made from numerous possible options. Highlighting the potential problem with researcher degrees of freedom, Simmons et al. (2011) showed that by selectively choosing specific variables in their models and also failing to report certain analysis steps, listening to the Beatles song ‘When I’m 64’ literally made participants younger – which is of course physically impossible. This demonstrated the ‘invisible multiplicity’ researcher degrees of freedom can provide (Gelman & Loken, 2013), as only the final analytical decisions tend to be reported but may not be the only analyses that were attempted. Given that most data sets can be generated and analysed in a number of different ways, and under the assumption, a type 1 error will be made ~ 5% of the time (if there is no true effect), a determined enough researcher would be able to demonstrate a seemingly real effect by applying a relatively small number of design/analysis variations to any given data set. For example, Carp et al. demonstrated at least 6912 different analysis pipelines, from ten different pre-processing steps for neuro-imaging data (Carp, 2012). This could potentially lead to 345 analyses that could obtain a false-positive result (*p* < .05).

One mechanism of combatting unfettered researcher degrees of freedom is pre-registration (Yamada, 2018), in which analyses decisions are publicly stated ahead of time (usually before data has been collected). However, this generally limits researchers to one a-priori-defined analysis, which may or may not be viewed by others as the most appropriate method. It is reasonable to suggest that for most data sets, researchers will disagree on how best to analyse them. Indeed, several ‘many analyst’ projects have demonstrated this case. Silberzahn et al. (2018) provided 61 analysts across 29 teams with the same data to address the research question of whether dark-skin-toned soccer players are more likely to be punished than light-skin-toned soccer players. They demonstrated considerable variability in the analyses. Sixty-nine percent of analyses found a significant effect (31% did not), with effect sizes ranging from odds ratio = 0.89 (slightly negative) to odds ratio = 2.93 (moderately positive). Importantly, the analysts were not motivated to demonstrate a ‘significant effect’ and accounting for their statistical expertise was unable to account for this variability. Similarly, Botcinik-Nezer et al. (2020) demonstrated that across 70 teams who analysed the data, no two teams chose identical analysis pathways for fMRI data, and there was considerable disagreement across teams for the tested hypotheses.

To overcome these issues, there has been a shift to multiverse analyses/specification curve analysis (SCA) to examine whether findings are robust to different analysis strategies (see Simonsohn et al., 2020; Steegen et al., 2016). In this case, the raw data which is collected for an experiment is used to construct a multiverse of data sets by combining different data processing decisions. Rather than report one single statistical analysis, all reasonable analyses are reported, as long as they are consistent with the underlying theory, statistically valid, and are not redundant with other specifications. These techniques have been used across psychology. For example, examining birth order effects on personality, with thousands of separate analyses (Rohrer et al., 2017). Similarly, using 20,004 (out of a possible 2.5 trillion) specifications, Orben and Przyblyski (2019) examined the link between well-being and technology usage in adolescents. As well as modelling variability based on analysis decisions, SCA help to identify trends (e.g. does the inclusion of specific covariates in models increase/decrease the effect) but also allow for an average effect size, based on all possible specifications (Flournoy et al., 2020).

Many published SCA analyses have examined the inclusion of different covariates into models, across multiple data sets. However, there has been little focus on whether pre-processing decisions, such as outlier removal taken can impact the magnitude of a given measure (outliers were addressed in the original paper describing specification curves: Simonsohn et al., 2020). These kinds of decisions are particularly important in cognitive/behavioural tasks, which measure reaction times and/or accuracy of responses. Outlier removal has been recognised as a potential data processing step which can impact findings (see Gress et al., 2018), and the reporting of outlier removal has increased during the replication crisis (Valentine et al., 2021). Often, there is no gold standard or widely accepted method of analysing data from these tasks. As such, there is considerable variability in their pre-processing across studies/lab groups which can impact their outcomes and reliability. For example, studies have shown that a priori decisions on the removal of outliers in reaction time distributions (e.g. using the mean vs. median, removing reaction times greater than 2 or 3 standard deviations around the individual mean) can impact the reliability of a widely used task to measure attentional bias (the Visual Probe task: Jones et al., 2018; Price et al., 2015), but also the Stroop and flanker tasks (Parsons, 2020), and contextual cueing tasks (Vadillo et al., 2023). However, other methods of removal also exist, such as transformation (e.g. recoding of extreme data points; Leys et al., 2019, provides a comprehensive overview of univariate outlier removal techniques).

The number of different decisions researchers might make when handling reaction time data is considerable and may have considerable impacts on theoretical and clinical findings. One research area where a large number of design and analysis decisions have been identified is the alcohol Stroop (see Jones et al., 2021). The alcohol Stroop is a widely used measure of ‘attentional bias’ in the addiction literature (Bollen et al., 2022). Attentional bias is the observation that individuals with an alcohol use history will show selective attention to substance-related cues in their environment, and this attention is thought to be indicative of current motivation to drink alcohol (craving), but also predict consumption (Field et al., 2016; Bollen et al., 2022). Furthermore, a line of research has attempted to target attentional biases as a candidate for psychological treatment for alcohol (mis)use (Fadardi & Cox, 2009).

Despite the widespread use of the task, findings are equivocal (for an excellent review see Bollen et al., 2022). Wider observations of the literature have also suggested that poor methodological practices should reduce any enthusiasm for positive results (Christiansen et al., 2015). Novel techniques such as specification curve analyses may help to resolve debates on the veracity of alcohol Stroop findings, but also identify any specific analyses pipelines which might increase/decrease any observed effects. Given the similarities in data-processing across different cognitive tasks, a focus on a well-established task with clearly identified specifications (Jones et al., 2021) may provide a useful template for how to analyse and report data from these tasks moving forward.

Therefore, the aim of this project was to conduct specification curve analysis on (1) previously published alcohol Stroop data, and also (2) novel data collected for this purpose. We also collected data on craving and alcohol use to examine whether different analytic decisions impacted correlations between Stroop interference and these outcomes. We decided to focus on the alcohol Stroop, given the widespread use of the task and its importance in testing theoretical predictions of attentional bias (Cox et al., 2006). These findings will inform us whether researcher degrees of freedom can impact the Stroop effect (as a measure of attentional bias) which may, in turn, impact our confidence in the task as a robust measurement tool in the addiction field.

## Methods

### The (alcohol) Stroop task

The alcohol Stroop task is a variant of the Stroop task (Cox et al., 2006). The standard Stroop presents colour names in different colours (e.g. the word ‘Red’ is presented in the colour ‘Blue’). Participants are asked to name the colour of the word whilst ignoring the semantic content. The inability to do this effectively is indicative of poor inhibitory control (Diamond, 2013).

The alcohol Stroop task uses two semantic-categories of words (alcohol-related and emotionally neutral) to generate a measure of attentional bias (the Stroop interference effect: reaction time to alcohol-related words – reaction time to neutral/comparison words). In line with the Stroop task, participants name the colour the words are presented in. Attentional bias is inferred through the difference in reaction times when alcohol-related words are presented compared to emotionally neutral words. For example, if it takes an individual longer to colour name alcohol-related words compared to the emotionally neutral words then they have an attentional bias to alcohol (i.e. their attention to the semantic content of the alcohol-related word means it takes them longer to identify the colour the word is presented in (Cox et al., 2006; Field & Cox, 2008). This paradigm has been used across various word categories to infer attentional bias towards or away from alcohol (see Fadardi & Cox, 2009).

## Stroop task data sets

### Previously published data

We re-analysed data from Spanakis et al. (2019). Specifically, we used data from an alcohol-related Stroop conducted via a mobile device using word stimuli. In this task, there were 66 critical trials, of which 33 were alcohol-related words and 33 were neutral/comparison words. There were 11 unique words in each category: alcohol (e.g. ‘pub’, ‘beer’, ‘wine’) and emotionally neutral (e.g. ‘bog’, ‘ravine’, ‘valley’). Each word was presented in three possible colours (blue, green, red). Each trial began with a central fixation dot presented for 500 ms. Following this, the alcohol or neutral word was presented with three response boxes underneath containing the colour names. The order of these response boxes was randomised on each trial. Participants were required to make a touch screen response by pressing the box with the correct colour name. Participants had 3000 ms to respond before the trial timed out and it was coded as an incorrect response. The task used a blocked design – one block contained only the alcohol-related words and a second contained the neutral words. Block order was randomised.

### Novel data set

We designed an alcohol Stroop to make it methodologically very different to the task utilized by Spanakis et al. (2019), to allow us to examine the robustness of analysis decisions across different task types. This is important, given that there are also considerable researcher degrees of freedom in how the alcohol Stroop is defined (Jones et al., 2021). The Stroop task was conducted online via a PC/laptop using a keyboard to record responses (rather than a touch screen).

The task was presented on a white background with a 300-ms fixation cross (‘+’) appearing in black before each word was presented. Words were presented in one of four possible colours; red, green, blue, or yellow, and participants were informed to ignore the content of the word and press a key indicating the colour. The key/colour information (D key for ‘red’, F key for ‘green’, J key for ‘blue’, K key for ‘yellow’) remained on screen at all times during the task. Participants first completed 24 practice trials with the words (‘one’, ‘two’, ‘three’,… ‘ten’) in different colours. To progress, participants had to register at least 80% correct responses on the practice trials. If participants failed to do so, the experiment ended, and participants were unable to re-attempt their participation. If participants made an incorrect response a red ‘x’ appeared for 400 ms.

Following the practice trials, there were 168 critical trials; 84 alcohol trials and 84 neutral trials, presented in completely random order. There were 14 alcohol-related words (e.g. ‘beer’, ‘alcopops’, ‘vodka’) and 14 emotionally neutral words (e.g. ‘box’, ‘queue’, ‘carpet’). If participants failed to respond after 3000 ms, a response was coded as incorrect. A completely randomised trial design was used (e.g. no blocks). In both tasks, the alcohol and emotionally neutral words were taken from previously established and widely implemented alcohol Stroop tasks (e.g. Fadardi & Cox, 2006), in which the emotionally neutral words have been matched for syllables, length and frequency within the English language, to control for any differences not related to the semantics of the word. This allows us to further generalise our findings to existing literature.

### Procedure for novel data set

Participants were recruited via Prolific (Peer et al., 2017), to participate in a study titled ‘*Cognitive Processes and Alcohol consumption*’ on December 10–11, 2020. The following Prolific screeners were used: aged 18+, resident in the UK, consumption of 14+ units of alcohol per week. Participants first read an information sheet and provided informed consent before providing demographic information (age in years and gender identity [male, female, non-binary, other]). Participants were then asked ‘*On a scale of 0 (no craving at all) to 100 (intense craving), how would you rate your craving for an alcoholic beverage at this moment in time*’, followed by the Alcohol Use Disorders Identification Task-C (AUDIT-C: (Bush et al., 1998) data not reported here). Following this, participants completed the Stroop task. After completing the Stroop task, participants were asked if they had been distracted at all during the task (yes, no) and debriefed. The experiment lasted approximately 10 min total, and participants were reimbursed £1.10. The Stroop task was programmed and presented using Inquisit Web version 5 (Millisecond Software, Seattle, WA. USA). We aimed to recruit > 119 participants as a power calculation determined this would be enough to detect a small difference in RTs between alcohol and neutral words (d = .23, based on Jones et al. (2021), with 80% power and alpha = .05). Available funds allowed us to oversample, and 166 participants were recruited. Ten participants did not make it past the practice trials.

## Specification curve and data analysis

For each specification curve, we begin with raw trial-level data from each participant on the alcohol Stroop task(s). First, all practice trials were removed, as is typical when calculating performance indices on these tasks. From the raw trial-level data, we recorded information on whether the trial was an alcohol-word trial or an emotionally neutral-word trial, and for each trial type, we recorded whether participants were correct (e.g. they identified the correct colour of the word) and the reaction time of their response. For each trial, reaction times on incorrect responses were not included when computing mean reaction times or standard deviations.

Following this initial raw data cleaning, we then passed this trial-level data through analytic code which determined each specification (e.g. removal of any reaction times > 4000 ms or removal of any reaction times > 3 standard deviations from the mean), before using the remaining data to compute a Stroop inference interference effect [RT to alcohol-related words – RT to neutral-related words]. This Stroop interference effect (in milliseconds) is our dependent variable for the main specification curves. Importantly, the specifications were not additive (e.g. the removal of any reaction times > 4000 ms, followed by the removal of reaction times > 3 SDs from the mean). Each specification was done in isolation on the individual trial data which had practice trials and reaction times from incorrect trials removed.

Our chosen specifications were taken from previous research which identified the different analytical approaches taken in the alcohol Stroop task (Jones et al., 2021). However, as these specifications themselves may only be present due to publication bias (for example, only specifications which led to a positive Stroop interference effect will be present in the published literature) we also identified two other measures of removing outliers discussed in Leys et al. (2019). These were the median absolute deviation, and Yuen’s trimmed means approach. Median absolute deviation it relies on the median as the estimate of the centre of the distribution, and on the absolute difference (rather than standard deviations: see Leys et al., 2013). Yuen’s trimmed means approach removes a percentage of extreme responses above and below the mean (here we used 10% and 20%) as cut-offs. The inclusion of these novel techniques would allow us to examine whether atypical techniques influence the overall Stroop effect.

Specification curve analyses are presented as a figure with an upper and lower panel. The upper panel presents the estimates and 95% confidence intervals of the Stroop interference effect for each specification ranked from smallest to largest. The lower panel presents a description of each specification grouped by type (see Table 1) to aid interpretation. Individual ticks in the lower panel correspond to the relevant estimate in the upper panel. We also include the intraclass correlation coefficients for effects of the data removal category (see Table 1) on the variability in the outcome (see Scharkow, 2019). This informs us how much variance in the Stroop Interference effect is explained by these categorical decisions.

**Table 1:** Analysis decisions for reaction times/errors based on the alcohol-Stroop task used in the specification curve

No exclusion	Individual RT removed / replaced based on SDs	Individual RT removal based on raw RTs	Participant removal	Median absolute deviation	Trimmed mean
No exclusions (median RTs used to calculate interference)	Removal of RTs > 3 SDs or < 2 SDs from individual mean	Removal of all RTS > 2000 ms	Participant removed if their mean RT > 4 SDs from the sample mean	Removal of RTS > 2.5 MAD from the individual median	Removing 20% of individual participants based on extreme values (from the mean)
No exclusions (mean RTs used to calculate interference)	Removal of RTs > 3SDs or < 3 SDs from individual mean	Removal of all RTS > 1500 ms	Participant removed if RT > 2 SDs from the sample mean	Removal of RTS > 2.5 MAD or < 2.5 MAD from the individual median	Removing 10% of individual participants based on extreme values (from the mean)
	Removal of RTs > 2 SDs or < 2 SDs from individual mean	Removal of all RTS > 1000 ms	Participant removed if number of errors > 3 SDs from the sample mean		
	Removal of RTs > 2 SDs from individual mean	Removal of all RTS < 400 ms	Participant removed if > 33% of errors on the task		
	Removal of RTs > 3 SDs from individual mean	Removal of all RTS < 300 ms	Participant removed if > 25% of errors on the task		
	Replacing RTs > 3 SDs from the mean with the mean	Removal of all RTS < 200 ms	Participant removed if Interference score > or < 4 SDS from sample interference score		
	Replacing RTs > 3 SDs from the mean with the mean + 3 SDs	Removal of all RTS < 150 ms	Participant removed if > 25% of RTS < 200 ms		
		Removal of all RTS < 100 ms			

To supplement the specification curves, we also examined the distribution of correlation coefficients of the alcohol Stroop effect when no outlier removal technique was used (in this case simply using the median reaction times) vs all other techniques (see Hussey, 2023). Considerable variability in correlation coefficients would suggest that choosing a different analytic pipeline would lead to different outcomes (and greater flexibility for selective reporting), whereas a narrow distribution of strong positive correlation coefficients suggests consistency in outcome irrespective of the analytic pipeline.

Raw data and analysis scripts can be found on OSF [https://osf.io/utnx2/]. Data were analysed in R using the ‘tidyverse’ (Wickham et al., 2019), and ‘specr’ (Masur & Scharkow, 2020) packages. Note, we are unable to share raw data from Spanakis (2019) as it is not permitted under the ethical approval of the original project. However, we share a synthetic version of the data using the r package ‘*synthpop’* (Nowok et al., 2016) for any users who may be interested.

## Results

### Published data

**Fig. 1:**
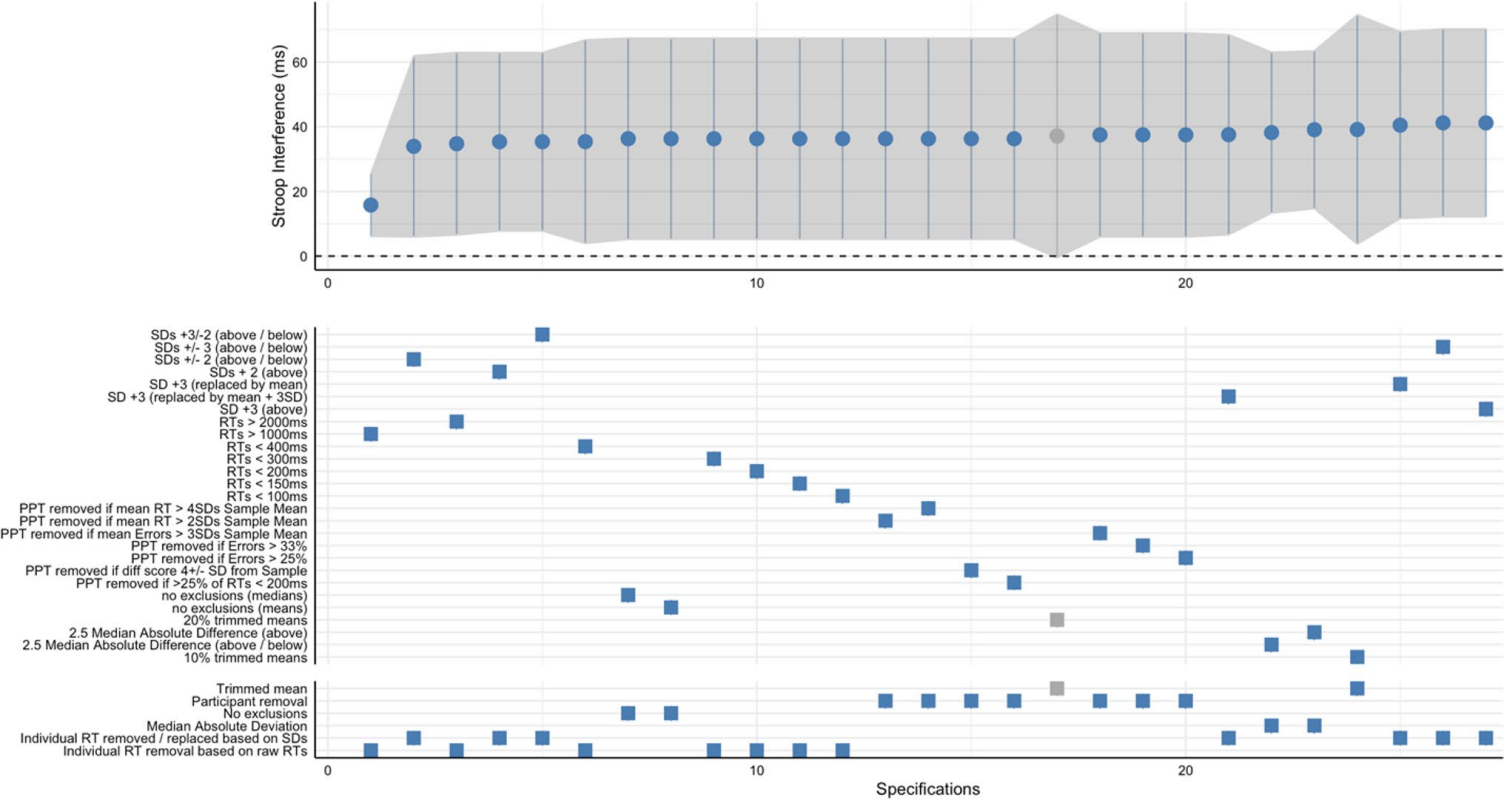
Specification curve for the analysis decisions of the Stroop data published in Spanakis et al. (2019). The outcome is the Stroop interference effect (difference in RTs for alcohol – emotionally neutral words)

Across the 27 specifications, the Stroop interference effect was robust, with a statistically significant positive score under all analysis decisions but one (20% trimmed mean). The median interference score was ~ 36.3 ms. The difference between the smallest and largest inference score was 25.4 ms (see Fig. 1 for the specification curve). The intraclass correlation coefficient of the data removal categories was 0.09, indicating about ~ 9% of variance in the Stroop interference effect estimates was explained by the categories.

## Distributions of correlation coefficients between no exclusions (median) and all other exclusions

**Fig. 2:**
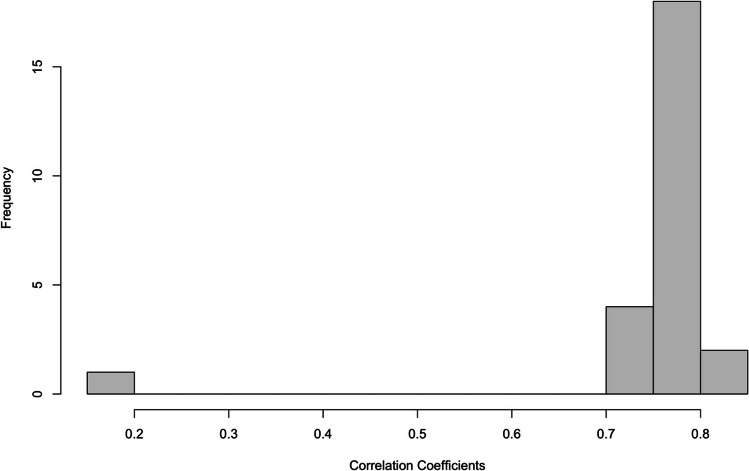
Distributions of the correlations of the alcohol Stroop interference effect when comparing no exclusions (median) to all other possible strategies, in the published data set

The distribution of correlation coefficients was narrow and clustered around strong positive correlations (mean *r* = .76, sd = .13; see Fig. 2). One correlation was clearly smaller (median ~ RTs > 1000) than the rest (*r* = 0.17). However, this suggests that there is a consistency in the alcohol Stroop effect *within* individuals irrespective of most exclusions.

## Novel data

### Stroop interference

**Fig. 3:**
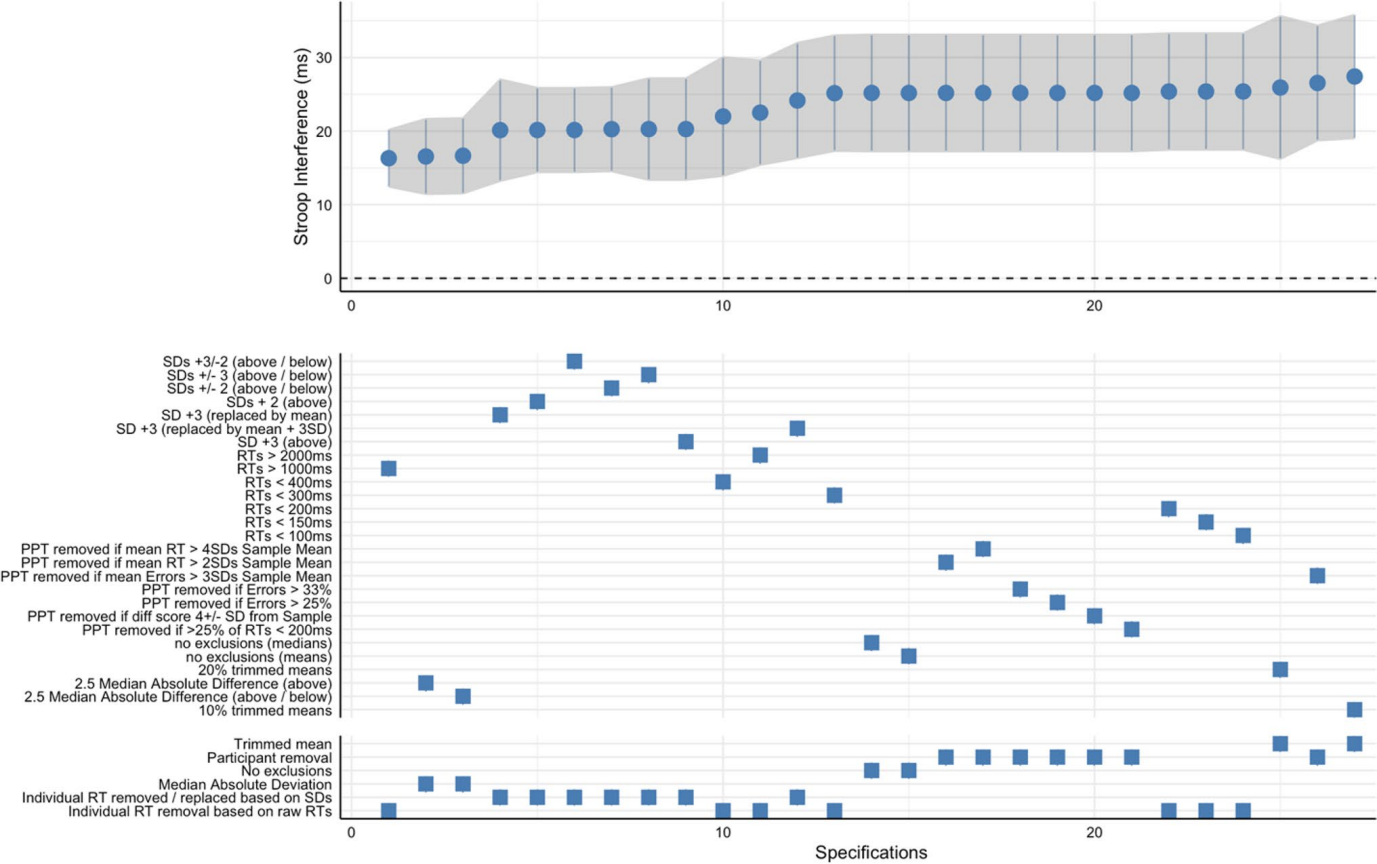
Specification curve for different analysis decisions of the novel alcohol Stroop data. The outcome is the Stroop interference effect (difference in RTs for alcohol – emotionally neutral words)

Across the 27 specifications, the Stroop interference effect was robust, with a statistically significant positive score under all analysis decisions (*p*s < 0.001). The median interference score was 25.2 ms. The difference between the largest and smallest Stroop interference score was 11.1 ms (see Fig. 3 for the specification curve). The intraclass correlation coefficient of the data removal categories was 0.74, indicating about ~ 74% of variance in the Stroop interference effect estimates was explained by the categories.

## Distributions of correlation coefficients between no exclusions (median) and all other exclusions

**Fig. 4:**
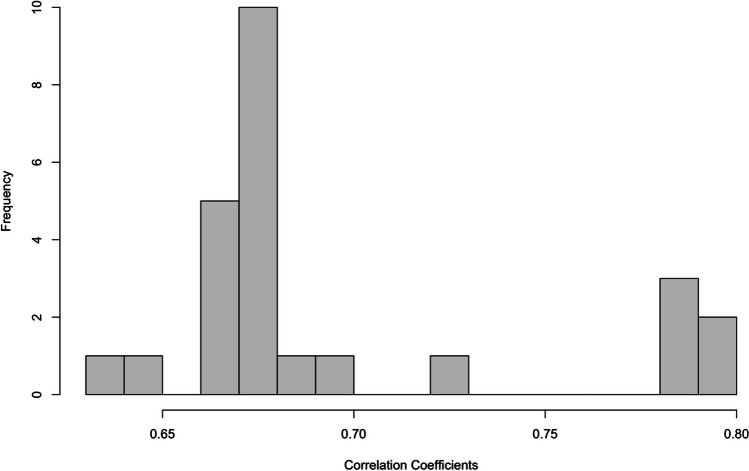
Distributions of the correlations of the alcohol Stroop interference effect when comparing no exclusions (median) to all other possible strategies, in the novel data set

The distribution of correlation coefficients was narrow and clustered around strong positive correlations (mean *r* = .69, sd = .04; see Fig. 4). In line with results from the published data, this suggests that there is a consistency in the alcohol Stroop effect *within* individuals irrespective of most exclusions.

## Discussion

In this study, we used multiple SCA to examine the effects of analytical flexibility (researcher degrees of freedom; Wicherts et al., 2016) on the outcome of the alcohol Stroop task. Across two data sets (one published, one novel), we observed some variability in the size of the Stroop interference score, however, the overall Stroop interference effect was statistically significant across all specifications in both datasets, except one. We also demonstrated some consistency of the effect intra-individually, when comparing different analytic pipelines to no exclusions. These findings (and methodology) will be of importance to scientists who use the alcohol Stroop in their research, but also to any researchers who might use similar reaction-time-based tasks and be faced with a multitude of potential analysis pipelines. This study also adds to the increasing evidence base of the usefulness of SCA. These methods are another tool researchers have against the replication crisis and in improving the rigour of their findings.

This is the first study to our knowledge to directly examine whether the flexibility in analysis decisions can influence a commonly used metric of attentional bias in addiction psychology. Our specifications were not arbitrary and based on previously identified analyses from a systematic review, and thus could be justified by researchers when analysing their own data. Furthermore, we also included specifications from other sources (Leys et al., 2019) to reduce the likelihood of only ‘successful’ specifications being present in previously published research. We demonstrated the robustness of the alcohol Stroop effect across a small but varied number of specifications. Our results also demonstrate that there was some variability in the effect sizes dependent upon analysis decisions, specifically when these decisions were based around outlier cut-offs using standard deviations from the individual mean and removal of upper bound reaction times. Removal of individual participants led to very little variability. This is likely because the criteria for removal (large number of errors) were strict. For example, Waters et al. (2009) demonstrated that < 1% of data were removed using these criteria. In our novel data, we likely constrained this further by removal of participants who made > 20% errors following the practice phase. Removal of individual RTs based on + 2/3SDs from the distribution generally led to smaller Stroop interference scores. It is likely that this is due to these cut-offs disproportionately remove longer reaction times to alcohol (rather than neutral) words. To our knowledge, when removing these ‘outlying’ reaction times, researchers tend to treat all reaction times as coming from the same distribution, rather than separate distributions for alcohol vs. neutral/control reaction times. However, if we assume a true Stroop effect, the overall distribution for alcohol reaction times should be different to that of neutral reaction times. The findings here support the alcohol Stroop interference effect as robust, and by extension theories that suggest drinkers have an attentional bias towards alcohol-related stimuli.

Only one specification led to a non-significant Stroop interference effect, in the published data. This was the 20% trimmed means approach. This specification requires the removal of the most data (from participants, rather than individual trial data). It is possible that this leads to a reduction of statistical power, as the overall estimate was similar to others; however the confidence intervals were considerably wider. Indeed, in the larger sample, the same pattern of results was not observed.

There are some limitations to our analyses. First, we focus on only the more prominent analysis decisions (identified in previous research; Jones et al., 2021) and as a result, our specification curves were relatively small. However, the overall pattern of results was consistent across all specifications for the Stroop effect. Furthermore, researchers have warned against overly inflating the analysis space with unnecessary specifications, which might serve to obscure reasonable effect estimates (Del Giudice & Gangestad, 2021). Secondly, our Stroop data were generated from atypical designs in which the Stroop was administered via a mobile device or online. Whilst psychology (and addiction science) transitions towards greater mobile and online testing (Gosling & Mason, 2015; Jones et al., 2022), these studies are still in the minority, making any generalisation to laboratory-based Stroop tasks more difficult (it is worth noting that our average Stroop interference scores were similar to interference scores in the lab: (Cox et al., 2006)). However, one might reasonably expect greater variability in reaction times when administered via mobile devices/online (Backx et al., 2020; Holden et al., 2019), making these data sets excellent candidates for our specification curves identifying more extreme responses. Nevertheless, future research should examine the full spectrum of possible specifications for the alcohol Stroop from data collected under laboratory-conditions to ensure the reliability of these effects.

In conclusion, we present the first SCA of the alcohol Stroop task. We chose reasonable specifications based on pre-existing analysis decisions as reported in a systematic review, as well as novel techniques not typically used in the field. The Stroop interference effect was robust but somewhat variable, supporting ‘attentional bias’ as a theoretical construct in alcohol use. We encourage researchers to consider the implementation of specification curve/multiverse analyses when analysing data from Stroop or similar cognitive tasks, to allow for the presentation of all possible analysis strategies. This will increase confidence in their findings, but also in the field moving forward.

## Data Availability

Raw data and analysis scripts can be found on OSF [https://osf.io/utnx2/].
